# A Production Calendar Based on Water Temperature, Spat Size, and Husbandry Practices Reduce OsHV-1 μvar Impact on Cultured Pacific Oyster *Crassostrea gigas* in the Ebro Delta (Catalonia), Mediterranean Coast of Spain

**DOI:** 10.3389/fphys.2017.00125

**Published:** 2017-03-03

**Authors:** Noèlia Carrasco, Ignasi Gairin, Josu Pérez, Karl B. Andree, Ana Roque, Margarita Fernández-Tejedor, Chris J. Rodgers, Cristobal Aguilera, M. Dolors Furones

**Affiliations:** Institute for Research and Technology in Food and AgricultureSant Carles de la Ràpita, Spain

**Keywords:** OsHV-1 μvar, Pacific oyster mortality, *Crassostrea gigas*, health management, culture strategies

## Abstract

Since 2006, the production of Pacific oyster *Crassostrea gigas* in the Ebro Delta area has dramatically declined from around 800 metric tons (MT) per year to 138 MT in 2011. This decline in production has had a significant socio-economic impact in a region where the shellfish sector is a traditional economic activity for many families. The identified agent responsible for this reduction in *C. gigas* production was Ostreid Herpesvirus microvar (OsHV-1 μvar), which has been associated with *C. gigas* spat mortalities in France, and in many other countries. In Spain the episodes of mortality became critical for the regional shellfish production between 2008 until 2014, with mortality percentage up to 100%. In this study, local hatchery *C. gigas* spat was used as sentinel animals for epidemiological studies and management tests carried out with the aim of reducing oyster mortality in the Ebro Delta area. A production calendar mainly based on water temperature dynamics was designed around an optimal schedule for spat immersion. The immersion calendar included two optimal periods for spat immersion, in summer when temperatures are ≥25°C and at the end of autumn and beginning of winter when they are ≤13°C. Such production planning has reduced mortalities from 80% (in 2014 and previous years) to 2–7.5% in 2015 in cemented oysters. Furthermore, other recommendations related to spat immersion size, culture density and methodology, and cementing calendar, which helped to achieve the results presented, were also recorded and transferred to local producers. This work presents a successfully tested management strategy reducing OsHV-1 μvar impact by designing new field management practices mainly focused on the handling and timing of spat immersion. This approach could be used as a management model in areas presenting similar production practices and environmental characteristics.

## Introduction

The ostreid herpesvirus microvar (OsHV-1 μvar) was described in 2010 as a virulent OsHV-1 variant associated with high mortality in spat and juvenile Pacific oyster *Crassostrea gigas* in France, at least since 2008 (Segarra et al., 2010), when temperatures reached 16°C. Since then, the problem of oyster herpesvirus has become a relevant issue in most of the European countries where Pacific oyster production occurs, as well as in other countries outside Europe, such as Australia and New Zealand (Jenkins et al., 2013; Keeling et al., 2014). France and New Zealand have approached this problem mainly with the development of selective breeding programs aimed toward virus-resistance oyster stocks in order to obtain better spat performance with higher survival when facing critical viral episodes (Dégremont et al., 2015a, 2016b; Pernet et al., 2016). On the other hand, health management has focused on optimal field practices and production strategies based on epidemiological studies, which could also be a relevant approach to reduce mortality. Regarding epidemiology, oyster antiviral response has been correlated with oyster age and water temperature, with 22°C being the critical water temperature value that induces the highest mortality in “*in vivo*” experiments (Green et al., 2014). The temperature threshold associated with disease outbreaks caused by the OsHV-1 varies according to study sites. In France recent studies reported temperatures between 16 and 20°C (up to 24°C) associated with disease outbreaks (Petton et al., 2013; Pernet et al., 2014), while in Australia the threshold for inducing mortalities was observed between 21 and 27°C (Paul-Pont et al., 2014). However, the important point seems to be the rapid temperature increase, passing by the 16°C threshold, until a virus peak is reached (Pernet et al., 2014; Renault et al., 2014; Barbosa-Solomieu et al., 2015). Horizontal transmission between different individuals, seems to occur when temperatures are between 16.2 and 21.9°C (Petton et al., 2013). Oyster age and size influences how the animals respond to an infection and ultimately effects survival rates (Dégremont, 2013; Green et al., 2014). Thus, both water temperature and oyster size seems to play a relevant role in OsHV-1 μvar epidemiology dynamics. In Australia, New Zealand and the French Mediterranean coast recent epidemiological studies have been carried out providing useful information for further management assays (Pernet et al., 2012; Paul-Pont et al., 2013a; Keeling et al., 2014; Whittington et al., 2015). Besides water temperature and oyster size, culture methodology including density of production and handling frequency, can also contribute to a reduction in pathogen impact (Pernet et al., 2012). Additionally, the management approach needs to be production-area specific taking into account the particularities of each site: the local epidemiology, and the environmental and production/commercial characteristics of each particular cultivation area (Pernet et al., 2016).

Spring mortalities of Pacific oyster (*C. gigas*) have been noticed by oyster farmers in the Ebro Delta Bays (Catalonia, Spain) since the beginning of the new millennium. However, in spring 2008 spat mortality increased dramatically, coinciding with the spread of the highly virulent ostreid herpesvirus microvar (OsHV-1 μvar; Segarra et al., 2010), and resulting with the first report of OsHV-1 μvar in Spain (Roque et al., 2012; Andree et al., 2014). Such events have been repeated since then every spring causing high economic losses for the sector. Pacific oyster production has decreased from 800 MT in 2006 to 138 MT in 2011, when this study started. Thus, production has significantly decreased in an area where bivalve farming is a relevant socio-economic activity. Because of this, oyster famers, research institutions, and autonomous government administrations, started working together focusing on an appropriate management of the disease, with the aim of protecting the future of the activity and ensuring economic sustainability. To this end the study objectives were: (1) production of virus-free local spat, for improving self-sufficiency of the local producers, and reducing the risk of pathogen introductions occurring with contaminated spat importations (historically sourced from the French Atlantic coast), (2) to define optimal field management practices to reduce oyster mortality due to the OsHV-1 μvar action and the final aim was making the activity sustainable and improve the situation regarding the problem of herpesvirus induced mortalities in the Ebro Delta production area. With these objectives, an experimental *C. gigas* local spat production program was initiated in Catalonia in 2011. The spat were used as sentinel animals in the epidemiological study and consecutive management tests. Production calendar and culture practices in the field, which could reduce the impact of herpesvirus were studied. Successful results, conclusions, and recommendations for producers are presented herein. The present approach could be interesting as a model for OsHV-1 μvar management in production areas with similar characteristics.

## Materials and methods

**Figure 1:**
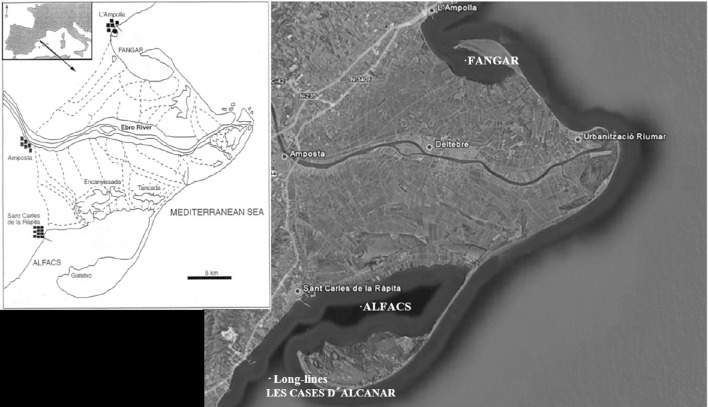
**Shellfish culture sites (Alfacs Bay, Fangar Bay, and Long-lines in open sea) situated in the Ebro Delta, in the Mediterranean coast of Catalonia, Spain**.

The experimental work was performed at IRTA experimental hatchery (Long, Lat: 0° 39′ 34.75″ E, 40° 37′ 39.95″N) and the Ebro Delta shellfish growing production areas of Alfacs Bay (Long, Lat: 0° 38′ 30.6619″ E, 40° 37′ 7.5023″ N), Fangar Bay (Long, Lat: 0° 44′ 48.8390″ E, 40° 46′ 20.8102″ N) and Alcanar (Long, Lat: 0° 34′ 10.9368″ E, 40° 30′ 30.8679″ N; Figure 1). Two parallel strategies were carried out:

### Experimental local spat production

In the Ebro Delta production areas there is no natural spat recruitment and producers traditionally import spat, mainly from France. To produce virus-free local spat each spring for use as sentinel animals 55.42–150 mm mature adult oyster specimens, which had survived one or two spring mortality episodes were collected from the field to be spawned by thermal induction in the IRTA experimental hatchery. Such approach was carried out between 2011 and 2014. In 2011, the first generation F1 of *C. gigas* spat was produced, F1 individuals were also produced in 2012, while in 2013 different F1 were produced, but also F2 from F1 specimens that had survived in the field for one/two spring mortality episodes. Different crossings, between these stocks were carried out following the same strategy in 2014, producing new F1 and F2. The long term aim was to mass select potential genetic tolerance to the virus over consecutive generations. In parallel, the experimental spat (F1 and F2 mix) was distributed to local producers to be used as a sentinel oysters for epidemiological studies and management tests. Previously the virus presence was screened, before leaving the hatchery and nursery, and before going to the natural bays. Thus, DNA was isolated from gills, digestive gland, and mantel tissues following DNeasy blood and tissue kit (Qiagen) and DNA quantity and quality measured with a GeneQuant spectrophotometer. Virus presence was screened by qPCR detection using the primers developed by Webb et al. (2007), detecting 10 pg (~50,000 genome copies)/50 μl (i.e., 200 fg/μl) of OsHV-1 DNA both in the absence and presence of *C. gigas* genomic DNA (15 ng/μl), or conventional PCR using primers C2-C6 (Renault et al., 2001), which have high sensitivity and can detect 1 fg of purified viral DNA, both with demonstrate effectiveness (Table 1). The methodology followed depended of the established protocol for the different projects under development at that moment by our team and their respective objectives. In order to not duplicate efforts and increase efficiency, those results were used for the present objective as they gave the information required.

**Table 1:** **Detection of OsHv-1 in broodstocks used for reproduction in IRTA experimental hatchery and spat obtained between 2011 and 2015**.

**Date**	**Lots**	**Size (mean) (mm)**	**Analyses**	**Result**
08.06.2011	Broodstock 1	82	qPCR	3+/30 = 10%
14.06.2012	Broodstock 2	84–150	qPCR	1+/30 = 3.34%
24.07.2013	Broodstock 3	131.45	qPCR	0+/30 = 0%
02.10.2013	Broodstook 4	55.42	qPCR	15+/27 = 55.55%
03.10.2013	Broodstock 5	73–32	qPCR	5+/30 = 16.67%
03.10.2011	Spat 1 (b1)	–	qPCR	0+/30 = 0%
31.01.2012	Spat 2 (b1)	20	PCR C2-C6	0+/30 = 0%
04.04.2012	Spat 3 (b1)	11–21	PCR C2-C6	0+/30 = 0%
04.12.2012	Juvenile 1 (b1)	30–45	PCR C2-C6	0+/30 = 0%
17.07.2013	Spat 4 (b2)	17.28	qPCR	0+/30 = 0%
21.01.2014	Spat 5 (b3)	–	qPCR	0+/30 = 0%
13.03.2014	Spat 6 (b3)	10–11	qPCR	0+/30 = 0%
28.05.2014	Spat 7 (b4 and 5)	11.89	qPCR	0+/30 = 0%
18.09.2014	Juvenile 2 (b4 and 5)	38	qPCR	0+/30 = 0%
26.11.2014	Juvenile 3 (b4 and 5)	44	qPCR	0+/30 = 0%
28.01.2015	Spat 8 (b4 and 5)	3.73	qPCR	0+/30 = 0%
28.01.2015	Spat 9 (b4 and 5)	18.70	qPCR	0+/30 = 0%

*b1, spat from broodstock 1; b2, spat from broodstock 2; b3, spat from broodstock 3; b4 and b5, spat from broodstock 4 and 5. PCR C2-C6 (Renault et al., 2001); qPCR (Webb et al., 2007)*.

### Local management practices to reduce mortalities produced by herpesvirus OSHV-1 μvar (spat immersion optimal calendar, spat size, culture method)

*C. gigas* F1 spat produced in 2011, 2012, 2013, and 2014 and F1+F2 produced in 2013 and 2014 were distributed to be used as a sentinel oysters between different producers between 2012 and 2015, in different locations of the Ebro Delta (Figure 1) in order to get epidemiological information to define an optimal production calendar based on water temperature dynamics. The objective was to define critical and optimal periods for spat immersion, when producers introduce and settle the spat in the cultivation area for further growing (optimal spat immersion calendar).

#### Epidemiological study

During 2011–2012, virus prevalences and mortality information of locally produced sentinel spat were recorded bimonthly in each of the three different geographical locations (Alfacs, Fangar, and Les Cases d'Alcanar) and graphically analyzed together with water temperature dynamics. From this study a potential optimal production calendar was designed.

#### Preliminary production calendar assays

Since 2011, when local hatchery spat started to be produced, information regarding mortality, immersion spat size (length), and culture methodology was recorded in order to register data on spat performance in the production area (Table 2). During 2013 and 2014, the first assays testing potential optimal periods for spat immersion were developed in collaboration with producers. To test the potential optimal periods for spat immersion, sentinel spat produced in 2012 and 2013 were immersed in the production area of Fangar Bay and their immersion size, culture methodology and survival followed during 2013 and 2014. Three different experimental spat immersions were carried out in collaboration with producer 3 in periods with low viral prevalences and oyster mortality identified in the previous epidemiological study (Table 2): (1) in June (spat size = 10–12 mm) when water temperature was 22°C, (2) in August 2013 (spat size = 6–8 mm), when temperatures reached and stabilized at 24–25°C, and (3) in February 2014 (spat size = 6–11 mm) during the pre-critical season, with colder temperatures around 13°C. When possible sampling (*n* = 30 individuals) was carried out in order to check the presence of herpesvirus associated to the mortality event observed in the batch (Table 2). DNA was isolated from oyster tissues following DNeasy blood and tissue kit (Qiagen) and virus presence was screened by qPCR (Webb et al., 2007) as described above.

**Table 2:** **Cumulative oyster mortality for producers that grown sentinel free-virus spat between end of 2011 and end of 2014, before the optimal production calendar and culture practices management were identified**.

**Producers**	**Spat size (mm)**	**Immersion date**	**Cultivation**	**Cum. Mortality (%) *(OsHV-1 qPCR result)***	**SEASON**
Prod. 1 (A)	T6-8	27/10/2011	Basket	80% *(24+/30 = 80%)*	AU
Prod. 2 (F)	T6-8	03/11/2011	Basket	90% *(7+/15 = 46.67%)*	AU
Prod 1 (A)	T6-8	22/03/2011	Basket	85%	SP
Prod. 3 (F)	T10-12	06/06/2013	Basket	100%	SU
Prod. 3 (F)	T6-8	12/08/2013	Cemented	40% *(8+/30 = 26.6%)*	AU
Prod. 3 (F)	T6-11	07/02/2014	Cemented	83% *(5+/12 = 42%)*	SP
Prod. 4 (F)	T5-7	07/02/2014	Fixed on shell	10%	SP
Prod. 4 (A)	T5-7	07/02/2014	Fixed on shell	80%	SP
Prod. 5 (A)	T6-12	12/03/2014	Basket	100% *(30+/30 = 100%)*	SP
Prod. 3 (F)	T10-14	03/06/2014	Cemented	80%	SU
Prod. 3 (F)	T6-12	08/07/2014	Basket	85%	SU
Prod. 3 (F)	T12-14	08/07/2014	Cemented	35%	SU
Prod. 6 (F)	T10-12	15/07/2014	Basket	70%	SU
Prod. 6 (F)	T 3-5	15/07/2014	Basket	10%	SU
Prod. 1 (F)	T 7-8	30/07/2014	Bags	60%	AU
Prod. 5 (A)	T10-15	31/07/2014	Bags	100%	AU
Prod. 7 (F)	T10-15	17/09/2014	Bags	80% *(15+/30 = 50%)*	AU
Prod. 6 (F)	T 3-5	24/11/2014	Basket	65%	SP

*Quantitative real time PCR (Webb et al., 2007) OsHV-1 results. SEASON refers to the mortality season; AU, autumn; SP, spring; SU, summer, A, Alfacs Bay; F, Fangar Bay*.

#### Testing experimental spat immersion calendar with optimal defined spat sizes and best performing cultivation methodology

The experiments were carried out between December 2014 and December 2015 in collaboration with producer 6 (Table 3) after evaluating the results from previous tests. Furthermore, optimal spat size range to be immersed, the seasonal timing of immersion, and best culture methodology performance inferred by field observations were selected as parameters to be approached in this study. Two lots of *C. gigas* local hatchery spat with sizes between 10 and 12 mm were immersed in the two previously defined potential optimal periods, in December after the Autumn virus episode when temperatures were <13°C and beginning of August when temperatures >25°C:

1. **The summer optimal period**, when water temperature is stabilized ≥25°C, in summer, usually from the end of July or beginning of August until the middle of September, in the Ebro Delta area. Spat size had to be at least between 8 and 12 mm. The limit of the period is correlated to the spat size. Spat size has to have sufficient time to increase in size to enable being cemented onto ropes by farmers at least 3–4 weeks before the virus critical period, when the water temperature passes by 16°C, during the autumnal cooling period, usually in the middle of November in the Ebro Delta area.
2. **The autumn/winter optimal period**, when water temperature is stabilized ≤13°C, usually from the beginning of December until approximately the end of January in the location of the Ebro Delta. Spat immersed in that period has to be between 8 and 15 mm. Immersion date and spat size are linked, since they have to be able to be cemented onto ropes at least 4–6 weeks before the virus critical period, before water temperature reaches 16°C, usually in the middle of April in the Ebro Delta area.

**Table 3:** **Cumulative oyster mortality for producers that grown sentinel virus-free 2014 spat between end of 2014 and end of 2015, following researcher recommendations for spat immersion calendar and culture management**.

**Producers**	**Spat size (mm)**	**Immersion date**	**Cultivation**	**Cum. Mortality (%)**	**Season**
Prod. 6 (F)	T10-12	05/12/2014	Cemented	6	SP
Prod. 8 (A)	T6-8	05/12/2014	Cemented	7.5	SP
Prod 6 (F)	T10-12	19/12/2014	Cemented	6	SP
Prod 6 (F)	T10-12	19/12/2014	Basket	20	SP
Prod. 7 (F)	T10-15	22/12/2014	Cemented	40	SP
Prod. 9 (A)	T14-15	08/01/2015	Cemented	2	SP
Prod. 1 (A)	T12-15	30/01/2015	Cemented	3.4	SP
Prod. 6 (F)	T12-15	10/07/2015	Cemented	2	AU
Prod. 6 (F)	T10-12	03/08/2015	Cemented	2	AU
Prod 6 (F)	T10-12	03/08/2015	Basket	5	AU

*SEASON refers to the mortality season; AU, autumn; SP, spring; SU, summer, A, Alfacs Bay; F, Fangar Bay*.

In each case, for each lot, oysters were cultivated comparing two different systems (basket and cemented). Mortality was recorded during all the study time especially before and after the virus events, as well as at the end of the production process. Mortality of other stocks (different origin, size, and cultivation method) cultivated in the same raft were also recorded for comparison (Table 4). Furthermore, other IRTA spat lots grown by other four producers were also surveyed during the study period (Table 3). Finally, spat origin, cultivation method, and mortality of non-IRTA spat grown by different producers were also recorded (Table 4). Mortality data for larger IRTA spat sizes (>20 mm) immersed was also surveyed, however those large sizes reached sizes higher than 35–40 mm at the time of virus episodes and were not critical regarding herpesvirus associated mortality and thus are not presented in the present manuscript.

**Table 4:** **Cumulative mortality (%) comparison between optimal managed sentinel virus-free spat and spat from other origins recorded in spring 2015 grouped by producers**.

**Producers**	**Spat size (mm)**	**Immersion date**	**Spat origin**	**Cultivation**	**Cum. Mortality (%)**
Prod. 6 (F)	T10-12	December 2014_1	SENTINEL	Cemented	6
Prod 6 (F)	T10-12	December 2014_2	SENTINEL	Cemented	6
Prod 6 (F)	T10-12	December 2014_2	SENTINEL	Basket	20
Prod 6 (F)	T20	February 2015	French TRIP	Cemented	15
Prod 6 (F)	T12-15	February 2015	French TRIP	Basket	65
Prod 6 (F)	T12-15	April 2015	French Natural	PVC tubes	35
Prod. 7 (F)	T10-15	December 2014	SENTINEL	Cemented	40
Prod 3 (F)	T6	January 2015	French Natural	PVC tubes	80
Prod 3 (F)	T6	January 2015	French Natural	Fixed on shell	80
Prod. 8 (F)	T20	May 2015	French Natural	PVC tubes	35
Prod. 8 (A)	T6-8	December 2014	SENTINEL	Cemented	7.5
Prod. 8 (A)	T8-10	February 2015	French Natural	PVC tubes	35
Prod. 10 (A)	T6-8	March 2015	French Natural	PVC tubes	70
Prod. 11 (A)	T10	February 2015	French Natural	PVC tubes	30
Prod. 9 (A)	T14-15	January 2015	SENTINEL	Cemented	2
Prod. 1 (A)	T12-15	January 2015	SENTINEL	Cemented	3.4

*TRIP, triploid oyster; A, Alfacs Bay; F, Fangar Bay. Gray values correspond to lowest mortality values recorded*.

## Results

### Experimental local spat production (virus-free hatchery and nursery, future perspectives of disease tolerance)

The production of *C. gigas* spat in the experimental hatchery developed at the IRTA facilities, had consecutive production of 101.100 units in 2011, 31.500 units in 2012 and ~1 million units, in both 2013 and 2014. Herpervirus was detected in four of the five bootstrap lots (5 × *n* = 30, total *n* = 150; see Table 1), collected from the Ebro Delta area and used for reproduction between 2011 and 2013, with recorded prevalences of infection between 3.34% (1+/30) and 16.67% (5+/30). Notably, in one of the batches, 55.56% (15+/27) prevalence was also recorded. However, no detection of the pathogen was recorded in the twelve quality control analyses of the produced spat (12 samples × *n* = 30 individuals; total *n* = 360), studied between October 2010 and January 2015.

### Epidemiological study

**Figure 2:**
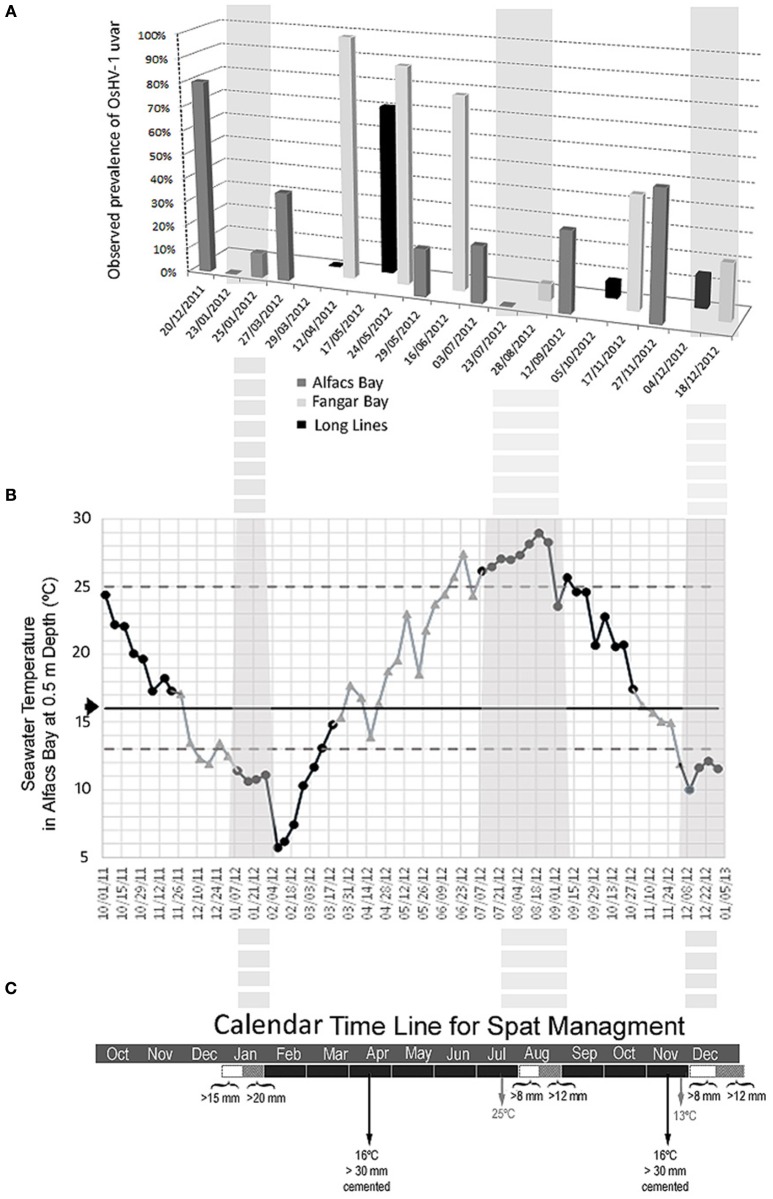
**(A)** Epidemiological study. Herpesvirus prevalence values (% of infected animals) in sentinel virus-free *C. gigas* spat cultured at three different sites in the Ebro Delta area (Alfacs Bay, Fangar Bay, and Long-lines in open sea) between end of 2011 and end of 2012. **(B)** Temperature dynamics in Alfacs bay between end of 2011 and end of 2012 were the more risky viral periods, the ramp of increasing temperature (16–25°C) and ramp of decreasing temperature (16–13°C) are in gray color, while the low virus prevalence periods are in black color. Texture boxes are highlighting the optimal periods (in autumn/winter and summer) for spat immersion having in account both, temperature dynamics and production planning for oyster growth. **(C)** Production calendar designed and tested between the end of 2014 and 2015, showing optimal periods for spat immersion in the Ebro Delta.

The epidemiological study carried out in 2012, in three different production locations of the Ebro Delta (Figure 1), showed the presence of OsHV-1 μvar throughout the year (Figure 2A). Herpesvirus OsHV-1 μvar 90% prevalence peaks, coinciding with high mortality values (85%), could be observed in spring when temperatures exceeded 16°C (Figure 2A). Furthermore, a second peak (50–80% virus prevalence) was observed in autumn when temperatures began to decrease passing again through the 16°C threshold (Figure 2A). High mortalities reaching 80% were also observed associated with low water temperatures (11.8°C) in December 2011 (80% virus prevalence), just after passing by 16°C in a dramatic decrease. However, low values of virus prevalence (6.67–33.34%) were observed between June and September, in summer when temperatures were ≥22°C, and between December and January at the end of autumn when temperatures were ≤13°C (13.34%). These periods were potentially interesting for oyster spat immersion and the starting of the oyster production in the field. Thus, from those studies potential optimal periods for spat immersion to allow an optimal production calendar in the Ebro Delta area were identified (Figures 2B,C).

### Preliminary production calendar assays

The first “optimal period” studies carried out in 2013/2014 showed that 10–12 mm spat immersed in June 2013, after showing negative result in qPCR (Webb et al., 2007), when water temperature was 22°C, suffered high mortality (80%) only 10 days after immersion of spat in the field (Table 2). In the second case, when spat was immersed in the field in August 2013 (size: 6–8 mm), when temperatures exceeded 25°C, the cumulative mortality recorded between the time of immersion and after the autumn viral episode was around 40% (Table 2) with 26, 67% of virus prevalence, and a 45% cumulative mortality after the following spring virus episode (oysters having reached 89 mm in size). In contrast, February 2014 immersed spat (size: 6–11 mm), cemented in March, experienced mortalities in April up to 83% (42% virus prevalence) during the critical period for the virus action (Table 2). From these results, it could be inferred a potential optimal period for spat immersion in summer when water temperature is stable at ≥25°C. However, no clear information was obtained to identify the adequate range for the optimal autumn/winter spat immersion time since the recorded mortality for the lot immersed in February 2014 was excessively high.

### Testing experimental spat immersion calendar with defined optimal spat sizes and best performing cultivation methodology

Taking into account previous results a better adjusted spat immersion calendar including optimal periods for spat immersion was re-designed and further tested (Figure 2C). The two spat lots studied, immersed on the 19th December 2014 and 3rd of August 2015 (with producer 6) showed low cumulative mortality values after the periods of herpesvirus impact (spring period usually with higher impact, and autumn period), ranging from 2 to 6% (Table 3), in comparison with previously recorded data (Table 2). The immersions were done during a potentially good temperature range taking into account the virus life cycle (water temperatures >25 and <13°C). In both cases, the immersion size (length) of the spat was 10–12 mm, to provide sufficient time in each case for growing to a size at which they could be comfortably cemented onto a rope (~4–6 weeks before the critical temperature for virus infection). From field observations, cemented spat showed better survival than other culture systems during the OsHV-1 episodes. For that reason this was the culture methodology selected during this study. In the Ebro Delta culture site cementation should be performed by producers usually before the end of February and end of October, respectively. Producer 6 in charge of the experiment also took care of the densities while oysters were in baskets before cementing onto ropes. Mortality values remained low until the end of the production. In comparison, spat immersed in December 2014, but cultured in baskets, suffered a 20% mortality after the virus critical period in spring (Table 3). Spat immersed in August 2015 and cultured in baskets, was maintained at low densities (125 units/basket box) and suffered a 5% mortality after the 2015 autumn viral episode. The other producers who also immersed the sentinel spat (local virus-free hatchery spat) in the defined optimal temperature periods and followed the instructions given for cementing onto ropes also recorded low mortality rates (2–7.5%), in both bays, throughout the virus episodes. Only one producer collaborating in the study (producer 7; Table 3) registered values of mortality higher than 40% in Fangar bay.

When studding the mortality recorded for non-local produced spat for the same period, we observed around 35% mortality in naturally collected French spat cultured on PVC tubes in both bays (producers 6, 8, and 11, Table 4). Some producers (3 and 10) working at both bays (Alfacs and Fangar) recorded up to 70–80% mortality using naturally collected French spat cultured on PVC tubes and fixed on shells. Triploid oyster also showed higher mortality than locally produced diploid spat (15% in cemented vs. 65% in basket).

## Discussion

Water temperature dynamics was used as a basis to define an optimal production calendar in the Ebro Delta reducing OsHV-1 μvar impact on *C. gigas* cultures. The epidemiological study showed that OsHV-1 μvar seems to be present during all seasons, in the Ebro Delta (at the three different locations: Alfacs Bay, Fangar Bay, and open sea), reaching high prevalence levels and causing high mortality rates when temperatures are more unstable, especially when they are increasing between 16 and 25°C in spring and summer. Similar results were observed in an integrative epidemiological study recently carried out in the Ebro Delta production area (Rodgers, personal communication). A second prevalence/mortality peak could be as well-observed when temperatures decreased in autumn, from 16°C until 12–13°C. A high mortality rate associated with high prevalence of the pathogen was observed once the water temperature was 11.8°C, just after a dramatic temperature drop from 16°C in December 2011. Each geographical location has a particular pattern to the virus epidemiology highly dependent on its local water temperature dynamics and possibly other water parameters. In Thau lagoon (North Mediterranean, France) two peaks of herpersvirus related mortalities were also observed usually occurring in May and September (Pernet et al., 2012), while in the Ebro Delta this usually occurs in April and November. The timing difference is probably linked to the difference in water temperature dynamics of the two locations, since Thau Lagoon is situated in a northern latitude and has its particular hydrodynamics. Taking in account the results the water temperature dynamics in our area a production calendar including best timing for spat immersion was determined and tested. An optimal period for spat immersion when water temperature stabilized >25°C, usually around beginning of August in Ebro Delta region was identified. Similar results where no oyster mortality was observed when water temperature was >24°C has also been reported in previous studies carried out in France (Pernet et al., 2012). Spat immersed in August 2013, with size around 15 mm and cultured by cementing to ropes showed higher survival during autumn and spring viral outbreak episodes (Table 2). The cementing system production reduces the density and stress during the oyster culture, and thus, the mortality due to herpesvirus action. Similar observations have been found in Thau lagoon in France, where mortality rates can vary from 30% (cement) to 80% (Australian baskets) depending of the culture system (Pernet et al., 2012). Later, experimental *C. gigas* spat of 10–12 mm immersed in August 2015 showed high survival results (95%), corroborating 2013 preliminary results. Furthermore, this spat was maintained throughout the experimental period at optimal densities (culture in basket, 195 animals per basket section, or cemented, 3 oysters each 20 cm) and non-stressed during the critical period. Thus, using experimental *C. gigas* sentinel spat of 10–15 mm immersed in August 2013 and 2015, kept at low densities until cemented to ropes, with the process of cementing performed at least 4 weeks before the autumn virus episode and not manipulated during the critical period successful survival resulted during autumn and spring consecutive mortality episodes (Tables 2, 3). Further studies should include complementary data on herpesvirus prevalence and infection intensity associated to survival/mortality values, helping to better understand host/pathogen interaction. Regarding optimal period for spat immersion when the water temperature is ≤13°C, the high mortality values of animals immersed in February 2014 are probably related to the fact that the spat probably had not achieved the optimal age/size (they were still 6–11 mm) to survive the virus action during the 2014 spring mortality event. Both, size and age are a critical factors conditioning oyster mortality caused by herpesvirus, since, age is correlated with immune system maturation and better performance against the virus (Dégremont, 2013; Green et al., 2014). Furthermore, that lot was probably cemented proximal to the expected spring viral outbreak event, and thus the oysters were both stressed and too small to achieve good survival.

Several experiments of spat immersion (spat size = 8–15 mm) were also carried out during December 2014 and January 2015 to further test the optimal spat immersion period when water temperatures are ≤13°C. These experiments contributed to better identify a second optimal period (autumn/winter) for spat immersion when the virus seems to be inactive and stable in cold temperatures below 16°C and around 13°C in winter. In most of the cases, the mortality observed was very low, between 2 and 7.5% (Table 3), when oysters were kept in low densities and cemented at least 4–6 weeks before the spring virus episode. While the lot showing 40% mortality probably had high oyster densities and was cemented late in the season taking in account the comments of the producer. It seems the cementing to ropes for this lot was carried out primarily in April 2015, when the water temperature was close to 16°C, just at the moment when the virus starts to be active and in which the oysters were, thus, stressed. Oysters cultured in baskets experienced a 20% mortality even following the optimal calendar management and optimal spat size for immersion, demonstrating that the cemented oysters gave better results. Thus, the second optimal period for spat immersion was identified after the autumn virus episode when the water temperatures decreased below 16°C and reached stability for the winter of ~13°C. Usually in the Ebro Delta bays, the autumn critical period is in November and the water temperature stabilizes around 13°C beginning in December. Thus, December and January seem to be good periods for 8–15 mm spat immersion. Small sizes, around 8 mm are better for immersion early in the optimal period, while in later immersions larger spat sizes give more guarantee of survival assuring that their being cemented to ropes occurs at the right time (4–6 weeks before water temperatures reach 16°C). In the Ebro Delta, the optimal moment for cementing of small spat immersed after the autumn virus event is at the end of February until beginning of March. Sizes larger than 20 mm, immersed at the end of January, and promptly cemented also had good survival results (Carrasco, data not shown). Regarding cold temperatures and absence of oyster mortality associated to herpesvirus, recent experiments have demonstrated that mortality of oysters infected with herpesvirus can be greatly reduced at 10 and 13°C (Pernet et al., 2015); however, the virus does not disappear and can be reactivated when temperatures increase again until 21°C producing high mortalities. Further recent observations have shown that viral transmission among oysters, in absence of mass mortality, can also occur when water temperature is below 16°C (Renault et al., 2014; Barbosa-Solomieu et al., 2015).

After autumn-winter spat immersion oysters grown during the winter season increased sufficiently in size and age (immune maturity). That fact, together with low culture densities (cementation) can allow better survival when temperatures rise again above 16°C and the viral activity is reactivated. The oyster production calendar traditionally used by local producers in the Ebro Delta included imported naturally recruited *C. gigas* spat (<8 mm) from France which were immersed in local waters during the months of November and February. Furthermore, those oysters are mainly cultivated in PVC tubes, with high densities, and held in a fixed raft inside the bays. Only a few weeks later, they had high mortalities coinciding with the autumn and spring virus episodes, respectively. Spat size, origin (area endemic for herpesvirus), cultivation methodology with high densities and transport and manipulation stress was the correct combination favoring virus replication and thus high mortality episodes. In recent years, some of the producers also started to try some assays of spat immersion after the mortality peak of the spring virus episode, in May and June, when water temperature were between 20 and 23°C. However, observed results were not optimal and moderate/high spat mortality was also recorded probably because water temperature was still below 25°C, and therefore, within the temperature range for virus activation and inducing oyster mortalities. Furthermore, other variables, more difficult to identify, such as the raft location inside the bay and thus, particular water currents surrounding the raft site could potentially also play a role in the distribution of mortality events (Pande et al., 2015). The patchy and clustered distribution of herpesvirus oyster mortality has already been reported and potentially linked with local hydrodynamics and herpesvirus being transported by planktonic particles (Paul-Pont et al., 2013b). It seems that the location of the oysters within the water column could also be associated to differences in virus effects and oyster mortality (Paul-Pont et al., 2013b). Clustered distributional patterns of herpesvirus associated mortalities in the production area have also been reported by recent observations in Thau lagoon where spat mortality was associated to energetic reserves and food quality available for the animals (Pernet et al., 2014). In the same study availability of nanophytoplankton was the most important variable for natural spat fixation in Chinese collectors. Knowledge of the pathogen and host physiology can be very useful in health management programs assuring the sustainability of the activity. Looking to potential genetic selected tolerance, taking in account that the mass selected local hatchery spat was F1 +F2 (up to two generations), and results of efficiency in OsHv-1 resistance selection has been observed after four generations of selection (Dégremont et al., 2015b), authors attribute the reduction of mortality mainly to the culture management strategies used during the study. Regarding ploidy, similar levels of virus infection and mortality were observed in both, diploids and triploids when selected for resistance (Dégremont et al., 2016a), however field observations suggest higher mortality levels in fast growing animals (Pernet et al., 2016), as is the case of triploids. This could explain the mortality registered over the study time in triploid lots.

In conclusion, the integrated management approach presented here focused mainly on a local oyster production calendar based on water temperature dynamics, but also taking into account culture methodology and spat immersion size. Such an approach seems to be an efficient low cost solution for producers to reduce oyster mortality associated with OsHV-1 μvar in the Ebro Delta. Furthermore, the management approach can be used as a model for other production areas with similar ambient characteristics. Finally, local producers who have participated in this study are significantly satisfied with the obtained results.

## Author contributions

NC has directed the study and analyzed the results. IG has participate in spat production and contact with producers. JP has performed the spat production. MF has recollected and analyzed environmental data. The rest of the authors has participated in getting funds or samplings.

### Conflict of interest statement

The authors declare that the research was conducted in the absence of any commercial or financial relationships that could be construed as a potential conflict of interest.
